# High definition transcranial direct current stimulation as an intervention for cognitive deficits in Alzheimer's dementia: A randomized controlled trial

**DOI:** 10.1016/j.tjpad.2024.100023

**Published:** 2025-01-01

**Authors:** Christian LoBue, Hsueh-Sheng Chiang, Amber Salter, Shawn McClintock, Trung P. Nguyen, Rebecca Logan, Eric Smernoff, Seema Pandya, John Hart

**Affiliations:** a Department of Psychiatry, University of Texas Southwestern Medical Center, Dallas, TX, USA; b Department of Neurological Surgery, University of Texas Southwestern Medical Center, Dallas, TX, USA; c Department of Neurology, University of Texas Southwestern Medical Center, Dallas, TX, USA; d AT&T Memory Center, Baylor University Medical Center, Dallas, TX, USA; e School of Behavioral and Brain Sciences, University of Texas at Dallas, Dallas, TX, USA

**Keywords:** Neuropsychological tests, Neurodegenerative disorders, Alzheimer's disease, Transcranial electrical stimulation, Transcranial direct current stimulation

## Abstract

**Background:**

Recent disease-modifying treatments for Alzheimer's disease show promise to slow cognitive decline, but show no efficacy towards reducing symptoms already manifested.

**Objectives:**

To investigate the efficacy of a novel noninvasive brain stimulation technique in modulating cognitive functioning in Alzheimer's dementia (AD).

**Design:**

Pilot, randomized, double-blind, parallel, sham-controlled study

**Setting:**

Clinical research site at UT Southwestern Medical Center

**Participants:**

Twenty-five participants with clinical diagnoses of AD were enrolled from cognition specialty clinics.

**Intervention:**

Treatment consisted of high definition transcranial direct current stimulation (HD-tDCS) delivered for 20 min over the medial prefrontal cortex. Ten sessions of sham, 1 mA, or 2 mA stimulation were received.

**Measurements:**

Cognitive outcomes were measured at baseline, after the last HD-tDCS session, and 8-weeks post-treatment. The primary outcome was change in total learning and delayed recall on the Rey Auditory Verbal Learning Test (RAVLT) immediately post-treatment and at 8-weeks. Secondary outcomes included measures of language, processing speed, and executive functioning. A multi-stage approach was used to examine cognitive outcomes, which included evaluation of effect sizes, statistical effects, and rate of clinically meaningful responses.

**Results:**

In this pilot trial, no statistically significant differences on cognitive outcomes were found between sham and active HD-tDCS immediately post-treatment (*p's* > 0.05). However, moderate-to-large effect sizes were identified for enhanced RAVLT total learning (Cohen's *d* = 0.69–0.93) and phonemic fluency (*d* = 1.08–1.49) for both active HD-tDCS conditions compared to sham, with rates of clinically relevant improvement between 25 and 33%. Meaningful enhancement persisted to 8 weeks only for the 1 mA condition.

**Conclusions:**

Multiple sessions of HD-tDCS over the medial prefrontal cortex appears to have potential to produce meaningful cognitive enhancements in a proportion of patients having AD with improvements maintained for at least 8 weeks in some.

**Trial Registration Information:**

ClinicalTrials.gov (NCT05270408). Registered December 30, 2021.

## 1 Introduction

Treatments cleared for Alzheimer's dementia (AD) show promise to delay cognitive decline, but do not lessen the cognitive deficits already present. New treatments that could enhance cognitive functioning, and in turn, lead to improvements in quality of life would fill an unmet need. Although AD involves decline in multiple cognitive domains, episodic memory decline is the prototypical feature for many with AD, wherein learning and remembering new events and personal experiences are impaired [1]. Only fragments of information are able to be encoded, stored, and recalled [2]. As a result, assessment of encoding and storage of verbal-based memories is the most sensitive approach for detecting and characterizing problems in episodic memory functioning in AD [3]. Hippocampal atrophy is closely linked to the episodic memory deficits, but there are multiple brain structures and tracts in the functional network (e.g., Papez circuit) for the encoding, storage, and retrieval of episodic memories that show abnormalities in AD [4]. In theory, modulation of a node in the neural network, or a distal region having strong connections to the network, could enhance functioning of the neural network and lessen associated AD symptoms. Noninvasive brain stimulation is capable of modulating neural networks [5] and has received considerable interest as a possible new intervention for AD.

Transcranial direct current stimulation (tDCS) is a form of noninvasive brain stimulation shown to increase neuronal activity from hyperpolarization of the resting membrane potential to promote long-term potentiation and neuroplasticity [6]. Such effects were magnified and persisted for considerably longer durations after repeated stimulation [7]. Thus, multiple sessions of tDCS might strengthen neural networks to produce long-lasting cognitive improvements in disorders such as AD. Not surprisingly, tDCS has been widely investigated in clinical trials in AD (see [8] for a detailed review). Most applied the electrical current to the left dorsolateral prefrontal cortex or left temporal cortex. Either way, stimulation likely reached components of the neural network for episodic memory, as the electrical field would have diffusely coursed through cortical and subcortical regions. However, the optimal montage for tDCS is still undetermined and most trials examined tDCS effects on global cognition only, rather than episodic memory and other specific cognitive domains [8]. Among the few that did, the length of treatment, current intensity, electrode configuration, outcome measures, and findings were inconsistent. As such, whether tDCS can produce clinically meaningful, reproducible, and durable effects on prominent cognitive problems in AD is unclear [8].

**Fig. 1:**
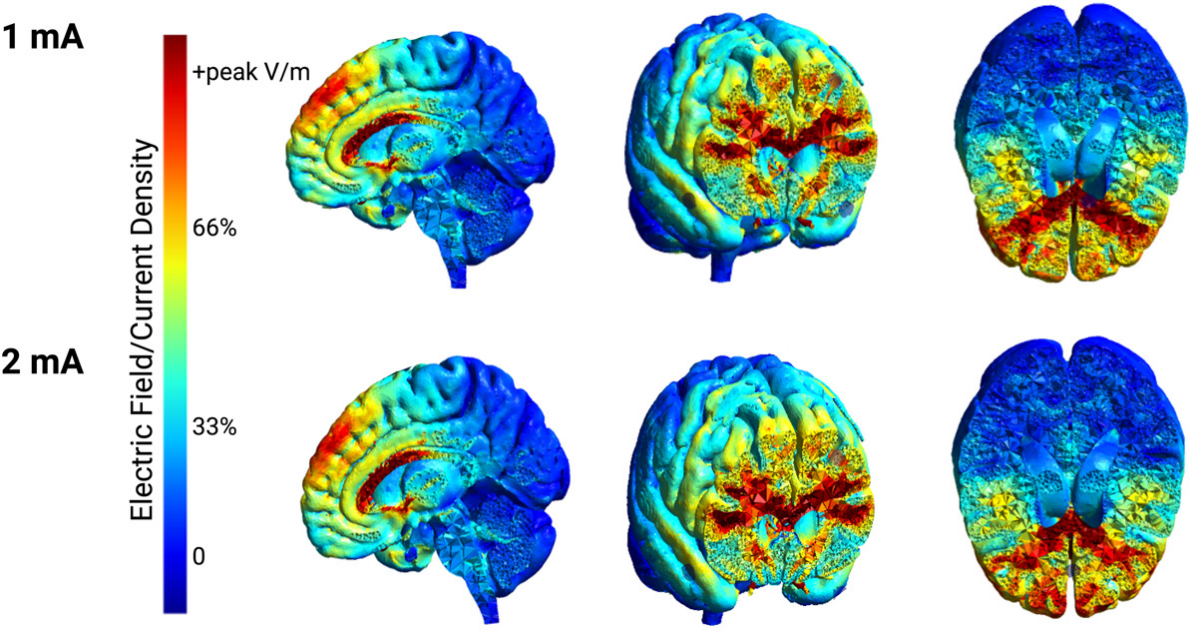
**Electrical Current Models During HD-tDCS**. Stimulation models using SIMNIBS of the active conditions with 1 mA and 2 mA current intensities. Computational modeling was performed on a multi-sequence average template brain for Alzheimer's dementia made available by Dadar et al. 2022 [38] and openly sourced at https://zenodo.org/records/5018356.

In light of these gaps, there continues to be a need to thoroughly examine the efficacy of tDCS on cognitive functioning in AD. High Definition tDCS (HD-tDCS) was developed to offer more focused control in delivering electrical stimulation than the conventional method used in nearly all prior AD trials. Consisting of small electrodes, there is more flexibility in positioning electrodes to apply stimulation with greater precision. The primary purpose of this pilot randomized controlled trial was to investigate if multiple sessions of HD-tDCS could lessen verbal episodic memory deficits in AD. Two current intensities (1 mA and 2 mA) were studied to examine dose-dependent effects given the intensity range of prior tDCS trials in AD and absence of empirical support for an optimal dosage to produce cognitive effects. The medial prefrontal cortex was chosen as the site for delivering the electrical current based on electrical field models indicating (Fig. 1) this was favorable for potential modulation of the dorsal anterior cingulate (dACC) as shown in prior HD-tDCS studies [9]. Because the dACC is part of a node in the neural network involved in episodic memory functioning [4,10], it was the region of interest to shape electrode configuration. However, to our knowledge, this target has not been explored in AD using HD-tDCS. The dACC and surrounding frontal structures and tracts are also involved in other cognitive abilities that decline in AD, including language, processing speed, and executive functioning [11]. Thus, a secondary aim was to examine these other cognitive domains as HD-tDCS induced outcomes. Duration of treatment effects was also explored by evaluating whether any modest cognitive changes produced from HD-tDCS persisted for 8 weeks.

## Methods

This pilot study was registered at Clinicaltrials.gov (NCT05270408) and included the recruitment of participants with a clinical diagnosis of possible or probable AD [12] from cognition specialty clinics in Dallas-Fort Worth. The trial was a randomized, double-blind, parallel, sham-controlled design. Participants received 10 daily sessions over a 2-week period of either sham, 1 mA, or 2 mA stimulation. Assignment was randomized by a computer at a ratio of 1:2:2 according to the sham (*n* = 5), 1 mA (*n* = 10), and 2 mA (*n* = 10) conditions. Participants and study personnel who performed outcome assessments were blinded to HD-tDCS condition. Cognitive assessments were completed at a baseline visit, immediately following the last HD-tDCS session, and again at an 8-week follow-up visit. The eligibility criteria, protocol, and statistical analysis plan are available in the **e-Materials**. The study was approved by the UT Southwestern Institutional Review Board and informed consent was obtained from all participants and their legal representatives.

### HD-tDCS protocol

HD-tDCS was delivered for 20 min on 10 separate days over a 2-week period using a Neuroelectrics Starstim® system with 12-mm Ag/AgCl disc electrodes (NE029-P.08.MD:GV) filled with conductive gel. Electrodes were placed in a 4 × 1 ring configuration on a Neuroelectrics neoprene headcap corresponding to the International 10–20 EEG system [13]. The anode was placed in the center at Fz (1 or 2 mA intensity) and surrounded by 4 cathodes at FPz, F7, F8, and Cz (each 25% of 1 or 2 mA). Active stimulation involved a 60-second ramp up period to the assigned current intensity (1 mA or 2 mA) and remaining at that intensity for 20 min. Sham HD-tDCS consisted of a 60-second ramp up to 1 mA and then turning off for 20 min. All HD-tDCS sessions were applied to participants while awake and resting.

### Cognitive measures

A brief cognitive assessment was completed and the primary outcome was the Rey Auditory Verbal Learning Test (RAVLT [14] as it is sensitive for measuring dysfunction of encoding and storage processes in verbal episodic memory in AD [15]. The RAVLT involves immediately recalling a list of 15 words read aloud over 5 consecutive presentation trials (encoding) and a 30-minute delayed recall trial (storage). Standardized scores were calculated to examine total learning (number of items immediately recalled for the 5 trials) and delayed recall based on widely used metanorms [16]. Age at baseline was used for deriving all standardized scores since an increase during the study could artificially inflate post-treatment scores, erroneously making performances better. Secondary outcomes included performances for visual episodic memory, language, processing speed, and executive functioning. Visual episodic memory was assessed through the Brief Visuospatial Memory Test-Revised (BVMT-R) [17]. Language was assessed with the Boston Naming Test (BNT) – 30 item form [18] (odd/even item versions; prorated for normative data [19] and Delis-Kaplan Executive Function System (DKEFS) Phonemic Fluency and Category Fluency [20]. Processing speed was assessed with the Southwestern Assessment of Processing Speed [SWAPS [21]], Trail Making Test Part A [TMT A [22]], and DKEFS Color-Word Interference Color Naming and Word Reading tasks [20]. Executive functioning was assessed with the Trail Making Test Part B [TMT B [22]], DKEFS Category Switching Fluency [20], and DKEFS Color-Word Interference Inhibition tasks [20]. Although negligible practice effects are exhibited with short retest intervals for AD, [23] alternate versions were used for multiple tests (RAVLT, BVMT-R, BNT-SF) to minimize potential for higher scores with repeated testing (see e-materials for more details on versions used).

### Statistical analysis

This pilot trial was designed as an exploratory study, and thus, was not powered to detect statistical differences between treatment conditions. As a result, a 3-stage approach was planned for thoroughly examining HD-tDCS effects on primary and secondary outcomes, which included characterization of effect sizes, statistical effects, and rate of clinically meaningful responses. Cognitive outcomes were examined between HD-tDCS conditions using general linear models (one-way analyses of covariance), with baseline performance entered as covariates. With two active HD-tDCS conditions and small sample sizes, two pairwise comparisons (not the omnibus test) for each measure were planned *a priori* and examined. This consisted of **a)** sham versus 1 mA stimulation and **b)** sham versus 2 mA stimulation. Eta^2^ effect sizes produced from the pairwise comparisons were transformed to Cohen's *d* for examination of effect sizes. Moreover, the proportion of individuals showing clinically relevant change were assessed in order to determine if HD-tDCS in this study's configuration was associated with meaningful cognitive enhancement or decline for each measure. Clinically meaningful change was defined as an increase or decrease of ≥ 5 points for standardized scores (T scores), which represents 0.5 standard deviations and a transition into a different performance category (e.g., switching from *mild-to-moderate impairment* to *mild impairment*, or vice versa). [19] For the exploratory objective of examining durability of a treatment effect, any outcomes that showed modest effects immediately post-treatment (statistical or moderate effect size with *d* ≥ 0.5) were assessed for persistence at 8 weeks. Statistical significance was defined as *p* < 0.05, with no correction for multiple comparisons given the small sample size of the pilot study.

## Results

### Characterization of sample

**Fig. 2:**
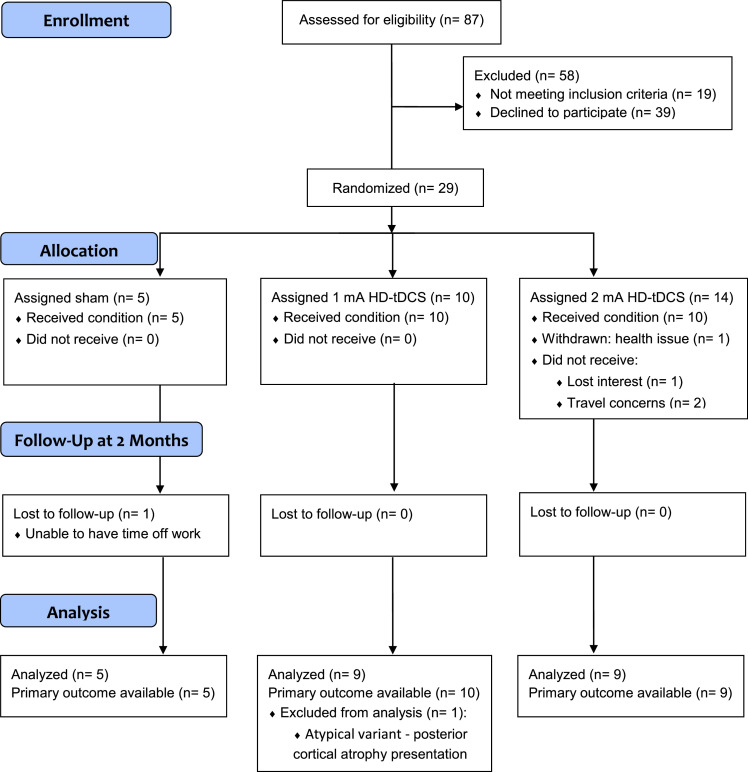
**Study Flowchart.** One participant assigned to 2 mA HD-tDCS had pre-existing atrial fibrillation, and cardiac decline was discovered mid-trial during a routine medical visit that required acute medical treatment and withdrawal; it was considered unrelated to HD-tDCS.

A summary of the recruitment and follow-up process is provided in Fig. 2. The characteristics across groups is detailed in Table 1. Time since AD diagnosis was a mean of 2.25 years (SD = 2.38) for the overall sample and the mean total score on the Everyday Cognition scale was 3.01 (SD = 0.58), representing a mild-to-moderate stage of AD [24]. Most participants were prescribed donepezil/rivastigmine (acetylcholinesterase inhibitor; 81%) and memantine (N-methyl-d-aspartate receptor antagonist; 57%) as treatment for AD symptoms, for which all had been stable for at least a month aside from one who started memantine the same week of receiving 2 mA HD-tDCS. Side-effects of HD-tDCS are detailed in the **e-Materials**. Standardized scores could not be derived for many participants on TMT B (*n* = 9) and DKEFS Color-Word Interference Inhibition (*n* = 9). The tasks were discontinued due to inability to complete practice items per administration rules or exceeding the time limit for completion of the actual test. Thus, data could not be examined for those two measures.

**Table 1:** Sample Characteristics.

		Sham (*n* = 5)		1 mA (*n* = 9)		2 mA (*n* = 9)	
Characteristic	Measurement						
Age	Mean ±SD	69.4	±9.0	74.0	±9.5	68.4	±7.8
Sex, Female	n (%)	2	(40)	4	(44)	5	(56)
Race, Non-White	n (%)	0	(0)	0	(0)	2	(22)
Ethnicity, Hispanic/Latinx	n (%)	0	(0)	1	(11)	0	(0)
Education, yrs	Mean ±SD	16	±3.2	14.3	±1.7	14.4	±2.4
Time since AD diagnosis	Mean ±SD	2.3	±1.0	3.4	±3.3	1.1	±1.0
ECOG total score	Mean ±SD	3.3	±0.6	3.2	0.6	2.7	0.4
ACTH inhibitor	n (%)	4	(75)	5	(56)	8	(89)
NMDAR antagonist	n (%)	4	(75)	4	(44)	4	(50)

ECOG = Everyday Cognition scale. ACTH inhibitor = acetylcholinesterase inhibitor. NMDAR antagonist = *N*-methyl-d-aspartate receptor antagonist.

### Blinding assessment

Participants and study personnel who performed assessments completed a questionnaire at the end of the study asking whether they believed active or sham HD-tDCS was applied or if they were uncertain. Less than 50% of participants (33–44%) and study personnel (22–25%) thought active HD-tDCS was received for the 1 mA and 2 mA conditions, and of those in the sham condition, 0–25% thought they received sham HD-tDCS, demonstrating successful blinding.

### Effect size results

A moderate-to-large effect size was demonstrated for higher RAVLT total learning immediately following the 10 treatment sessions for both active HD-tDCS conditions compared to sham (Cohen's *d* = 0.69 - 0.93), when adjusting for baseline performance. There was an average difference of ∼4 standardized points after 1 mA and 2 mA stimulation relative to sham (Table 2). For RAVLT delayed recall, only a moderate-to-large effect size for lower performance was found following 1 mA stimulation relative to sham, though the average difference was < 1 standardized score. A large effect size for higher DKEFS Phonemic Fluency was also seen for the 1 mA and 2 mA conditions relative to sham (Cohen's *d* = 1.08 – 1.49). The average difference was ∼8 standardized points for both, but driven in part by the sham group performing worse at retesting. From pre- to post-treatment, phonemic fluency improved by ∼3 standardized points after 1 mA and 2 mA HD-tDCS. For most other secondary outcomes, minimal effects were seen for 1 mA HD-tDS compared to sham, though a moderate-to-large effect size was seen for lower SWAPS and TMT A performance, 2 of the 4 processing speed measures. In comparing 2 mA HD-tDCS to sham, there were some moderate effect sizes that were equivocal. Effects for lower BVMT-R total learning, DKEFS Category Fluency and Category Switching, and TMTA were found for those receiving 2 mA HD-tDCS, but several were related to the sham group having better performance at retesting (see section on *Clinically Meaningful Responses*). In contrast, a moderate-to-large effect size was found for higher BVMT-R delayed recall following 2 mA HD-tDCS compared to sham, and no modest effects were found on the remaining language and processing speed measures.

**Table 2:** Cognitive Outcomes.

	Sham		1 mA					2 mA				
Measures (T scores)	Time 1	Time 2	Time 1	Time 2	Adj M Diff (T2 vs sham)	ES*	p	Time 1	Time 2	Adj M Diff (T2 vs sham)	ES*	p
RAVLT total learning	13.8	13.4	17.8	21.0	+ 3.9	0.93	0.25	18.0	21.9	+ 4.6	0.69	0.18
RAVLT delayed recall	20.6	21.2	24.4	24.0	- 0.7	0.74	0.48	22.3	22.3	- 0.4	0.19	0.64
BVMT-R total learning	20.8	22.8	24.2	24.2	0.0	0.03	0.98	21.7	21.1	- 2.0	0.86	0.22
BVMT-R delayed recall	21.4	20.6	26.9	24.7	+ 0.1	0.04	0.97	20.3	22.8	+ 2.9	0.69	0.23
BNT-SF	30.6	30.4	31.6	31.5	+ 0.2	0.04	0.94	30.1	31.9	+ 1.9	0.42	0.47
DKEFS phonemic fluency	39.0	33.6	37.5	40.4	+ 8.1	1.49	0.06	32.7	35.9	+ 7.8	1.08	0.07
DKEFS category fluency	24.2	25.0	28.1	30.9	+ 1.8	0.19	0.61	24.9	22.8	- 2.9	0.68	0.38
DKEFS category switching	20.0	21.6	25.3	27.4	+ 0.8	0.20	0.83	24.0	23.8	- 1.6	0.94	0.64
TMT A	33.4	38.8	34.7	35.1	- 4.9	0.56	0.28	29.4	31.3	- 3.7	0.51	0.42
TMT B	–	–	–	–	–	–	–	–	–	–	–	–
SWAPS	27.6	30.2	34.0	31.0	- 4.5	0.96	0.11	26.1	27.9	- 1.1	0.1	0.68
DKEFS Color Naming	25.0	31.4	32.2	36.0	- 2.7	0.20	0.61	24.5	28.4	- 2.5	0.24	0.63
DKEFS Word Reading	31.4	35.2	38.9	39.9	- 1.1	0.00	0.73	36.5	36.1	- 3.0	0.18	0.65
DKEFS Inhibition	–	–	–	–	–	–	–	–	–	–	–	–

Adj M Diff = Mean difference of post-treatment scores (Time 2; T2) between active HD-tDCS condition and sham adjusted for pre-treatment scores (Time 1) from general linear models. Measures of variability are listed in e-Table 2 of the e-Materials. ES* = Cohen's d effect size for mean difference of post-treatment scores between active HD-tDCS condition and sham adjusted for pre-treatment scores. *p* = *p*-value. RAVLT = Rey Auditory Verbal Learning Test. BVMT-*R* = Brief Visuospatial Memory Test-Revised. BNT-SF = Boston Naming Test 30-item Short Form (odd and even). DKEFS = Delis-Kaplan Executive Function System. TMT = Trail Making Test. SWAPS = Southwestern Assessment of Processing Speed.

### General linear model results

RAVLT total learning and delayed recall did not show statistically significant differences between sham and active HD-tDCS (1 mA or 2 mA) immediately following the 10 treatment sessions after adjusting for baseline performance (Table 3; *p's* > 0.05). Likewise, there were no statistically significant differences on any secondary outcome measure between sham and active HD-tDCS (1 mA or 2 mA) immediately post-treatment (*p's* > 0.05).

**Table 3:** Clinically Meaningful Change from Baseline to Immediate Post-treatment.

	Sham (*n* = 5)		1 mA (*n* = 9)		2 mA (*n* = 9)	
Measure	% Improve	% Decline	% Improve	% Decline	% Improve	% Decline
RAVLT total learning	0	20	33	11	33	0
RAVLT delayed recall	0	0	0	0	0	0
BVMT-R total learning	20	0	22	22	0	11
BVMT-R delayed recall	0	0	0	33	11	0
BNT-SF	0	0	12	0	22	0
DKEFS phonemic fluency	0	40	25	12	33	11
DKEFS category fluency	20	0	37	12	0	22
DKEFS category switching	20	0	25	12	0	0
TMT A	40	20	33	22	22	11
TMT B	–	–	–	–	–	–
SWAPS	20	0	0	22	37	12
DKEFS Color Naming	40	20	44	0	37	0
DKEFS Word Reading	40	20	22	11	12	25
DKEFS Inhibition	–	–	–	–	–	–

RAVLT = Rey Auditory Verbal Learning Test. BVMT-*R* = Brief Visuospatial Memory Test-Revised. BNT-SF = Boston Naming Test 30-item Short Form (odd and even). DKEFS = Delis-Kaplan Executive Function System. TMT = Trail Making Test. SWAPS = Southwestern Assessment of Processing Speed.

### Clinically meaningful change results

On the primary outcome, clinically meaningful improvement occurred on RAVLT total learning for 33% of participants receiving 1 mA HD-tDCS, 33% of participants receiving 2 mA HD-tDCS, and 0% receiving sham (Table 3). In contrast, some who had sham (20%) and 1 mA stimulation (11%) showed clinically meaningful worsening, but none with 2 mA HD-tDCS did. For RAVLT delayed recall, no individuals had clinically meaningful changes (improvement or worsening for any HD-tDCS condition).

On secondary outcomes, DKEFS Phonemic Fluency exhibited clinically meaningful improvement for 25% of participants receiving 1 mA HD-tDCS and 33% of participants receiving 2 mA HD-tDCS, whereas only a meaningful decline was shown in the sham condition (40%). However, some who had 1 mA (12%) and 2 mA (11%) stimulation showed a meaningful decline in phonemic fluency. Rates of clinically meaningful change (improvement or decline) were negligible (< 25%) for nearly all HD-tDCS conditions on BNT-SF, DKEFS Category Fluency and Category Switching, and BVMT-R total learning and delayed recall. The only exceptions were meaningful improvement on DKEFS Category Fluency for 37% and meaningful decline on BVMT-R delayed recall for 33% of participants receiving 1 mA HD-tDCS. On processing speed measures, rates of clinically meaningful improvement were 33–40% across most tasks for the sham condition, and an inconsistent pattern for the active HD-tDCS conditions. Rates of meaningful decline on processing speed measures were minimal aside from a single, modest decline found on DKEFS Word Reading for 25% of those receiving 2 mA HD-tDCS.

### Eight-week follow-up

Effects on RAVLT total learning (1 mA and 2 mA), BVMT-R delayed recall (2 mA only), and DKEFS phonemic fluency (1 mA and 2 mA) were the outcomes assessed for persistence out to 8-weeks. While no statistically significant differences were identified between sham and active HD-tDCS on RAVLT total learning (*p's* = 0.22 – 0.88), BVMT-R delayed recall (*p* = 0.62), or DKEFS phonemic fluency (*p's* = 0.14 – 0.73), moderate-to-large effect sizes were seen at 8 weeks post-treatment. Higher RAVLT total learning (Cohen's *d* = 0.74; M diff = +5.6) and DKEFS phonemic fluency (*d* = 0.88; M diff = +6.3) were found for 1 mA HD-tDCS relative to sham, with an average difference of ∼6 standardized scores when adjusting for baseline performance. Minimal effect sizes at 8-weeks post-treatment were seen on cognitive measures for 2 mA HD-tDCS compared to sham.

## Discussion

Memory impairment in AD involves the loss of the ability to encode and store information, and a key question has been whether neuromodulation could enhance such processes to lessen deficits. While this pilot trial was not powered to demonstrate statistically significant treatment differences, a modest effect for enhanced verbal learning was observed for both active HD-tDCS conditions (1 mA and 2 mA) compared to sham. Clinically meaningful improvement occurred in 33% of individuals receiving 1 mA and 2 mA HD-tDCS, whereas none did with sham. It is worth noting that these response rates are similar to the rates reported in many successful neuromodulation trials in neuropsychiatric disorders [[25], [26], [27]]. Robust verbal learning enhancement persisted out to 8 weeks, but only for the 1 mA condition. Although the results are encouraging and might indicate enhanced encoding or retrieval for some, there were no apparent treatment effects for delayed recall. Thus, a pattern of rapid and severe forgetting remained, suggesting no change in the limited information transferred into storage.

Only four prior trials to date have investigated the effects of multiple sessions of tDCS/HD-tDCS on episodic memory in AD [[28], [29], [30], [31]]. Three reported statistically significant enhancement, albeit on different measures consisting of total learning on a word-list memory task [29], a generalized index of episodic memory performance [30], and a composite score of delayed recall/recognition performance [31]. When merged with our results, multiple lines point to enhanced encoding for a selection of patients with AD following tDCS/HD-tDCS. Nonetheless, despite one prior trial finding storage processes in episodic memory were improved [31], it is unclear if the enhancement was in delayed recall, recognition, or both. If only recognition improved, how that translates to better real-world functioning is unclear, as recalling information without assistance (e.g., cues) would still be highly diminished. As it stands currently, storing and remembering information in episodic memory has not been shown to be amenable to neuromodulation in AD. Degradation of key structures in the neural network (i.e.., hippocampus) could be beyond alteration, leaving uncertainty about the clinical relevance if only learning and recognition are enhanced. A critical future research direction might be to determine if improvements in learning or recognition could produce a minimum clinically important difference [32] and change in everyday cognitive functioning in AD.

Even if the neural network for episodic memory may be too damaged to effectively modulate, there could be other cognitive deficits to target with neuromodulation therapies in AD. Despite the potential, data is lacking on tDCS/HD-tDCS effects on language, processing speed, and executive functioning in AD. In this pilot trial, an effect for enhanced phonemic fluency was observed for both active HD-tDCS conditions, wherein 25% of individuals receiving 1 mA HD-tDCS and 33% receiving 2 mA HD-tDCS had clinically meaningful improvement compared to none for sham. Meaningful enhancement even persisted out to 8 weeks for the 1 mA condition (*M* = 41.67), as the increase involved a transition from a clinical classification of *mild impairment* to the *low average* category at both time points after treatment. A change in processing speed could underlie an improvement, but unlikely since changes in processing speed were generally better for those receiving sham. With no effects seen on BNT-SF or category fluency, neither did language functioning broadly improve with HD-tDCS.

Word retrieval for tasks engage different neural networks. Phonemic fluency primarily engages frontal networks including the dACC while BNT-SF and category fluency rely more on temporal networks [33]. As such, it is conceivable that frontal networks were selectively stimulated and modulated with the HD-tDCS protocol, which was the cortical network we sought to engage. Neuroimaging tools (e.g., electroencephalography) are highly sensitive and valuable for identifying cortical network alteration [9,34], but in their absence, analyzing if multiple cognitive functions are altered in a shared network allows for localization. Executive functioning would be expected to be enhanced in this scenario. Yet, the single measure of executive functioning capable of being analyzed did not show improvement. An inability for many participants to complete TMT B and DKEFS Color-Word Interference Inhibition prohibited a thorough examination given standardized scores could not be derived. As a result, the implications and clinical relevance of enhanced phonemic fluency is unclear. Moving forward, different outcome metrics or alternative scoring indices for the measures of executive functioning will be necessary to evaluate if tDCS/HD-tDCS has treatment effects on this cognitive domain in AD.

Neuromodulation outcomes are found to vary in neuropsychiatric disorders [35]. The fact that some persons in this trial had meaningful enhancement and others meaningful decline suggests this variation also extends to AD. One prominent idea for the variation relates to use of a one-site-fits-all approach to tDCS/HD-tDCS. Nearly all trials in neuropsychiatric disorders and AD apply stimulation at one site for all persons based on electrode positions with the International EEG method. However, head shape, scalp and skull thickness, distance between skull and cortex, and volume of cerebrospinal fluid affect neuromodulation efficacy and are not accounted for with the method [36]. When considering the extent of cortical atrophy alone is heterogeneous in AD, leading to variation in skull-to-cortex distance and cerebrospinal fluid volume, applying stimulation at the same position across all individuals would be expected to produce different modulatory effects. New methods have been pioneered to employ neuroimaging data for personalization of tDCS/HD-tDCS protocols based on interindividual head/brain variations [35,36]. Increased precision could lessen variation in outcomes and lead to better treatment efficacy. However, a direct comparison of personalized and conventional strategies to stimulation are needed to understand if outcomes are more robust and reproducible.

This pilot study has several strengths, including its sham-controlled design, detailed cognitive data, and rates of clinically meaningful responses. Nonetheless, the study does have limitations beyond small sample sizes. HD-tDCS was delivered while participants were sitting comfortably at rest and there was no standardization of what constituted “rest,” as with many neuromodulation trials. Participants were free to sit silently so long as they were awake, interact with mobile devices, or converse with operators during stimulation. There has been some evidence that neuromodulation effects may depend on the cognitive state during stimulation, for which this study is unable to control or account for given nonstandardization [37]. Furthermore, a functional living metric of cognitive function was not collected as an outcome. As a result, the clinically meaningful responses identified could not be correlated with changes in daily living to establish a real-world functional change.

Delivering multiple sessions of HD-tDCS over the medial prefrontal cortex showed the potential to produce meaningful cognitive enhancements in a proportion of patients with mild-to-moderate AD. Deficits in verbal learning and phonemic fluency showed improvement from stimulation at 1 mA and 2 mA current intensities, but meaningful effects only persisted out to 8 weeks for the 1 mA condition. Minimal evidence currently exists for deficits in storing and recalling information in episodic memory to be amenable to neuromodulation in AD. At the present time, these preliminary results suggest that other areas of cognitive functioning such as language and/or executive functioning may be more promising targets in AD for neuromodulation.

## Declaration of competing interest

The authors report no disclosures relevant to the manuscript beyond the funding information listed above.

## Appendix Supplementary materials

Image, application 1

Image, application 2

Image, application 3
