# Supplementary material for: High definition transcranial direct current stimulation as an intervention for cognitive deficits in Alzheimer's dementia: A randomized controlled trial
Source: J Prev Alzheimers Dis. 2025 Jan 1;12(2):100023. doi: 10.1016/j.tjpad.2024.100023 (PMC11895854; doi:10.1016/j.tjpad.2024.100023)
Supplement: Supplementary file 1 [file mmc1.docx]

**Supplemental Materials**

**Setting, Dates of Enrollment, Reason Trial Ended**

**Eligibility Criteria for Participants**

**Headcap Positioning for HD-tDCS**

**Clinical Information Collected**

**Method of Deriving Cognitive Scores and Test Forms Used**

**Method of Evaluating 8-week Follow-up Data**

**HD-tDCS Tolerability**

**e-Table 1 Stimulation Side Effects and Tolerability**

**e-Table 2 Measurement Variability on Cognitive Outcomes**

**e-References**

**Setting, Dates of Enrollment, Reason Trial Ended**

The study setting was a clinical research site at UT Southwestern Medical Center. Enrollment began July 13, 2022 and ended December 5, 2023. The trial was completed when 25 AD participants were allocated to and received an HD-tDCS condition.

**Eligibility Criteria for Participants**

Inclusion/exclusion criteria consisted of: age 50 years and older, English proficiency, absence of metallic fragments in head, absence of a substance use disorder within the past year, and absence of a major medical/psychiatric condition that could either obscure results (e.g., traumatic brain injury < 1 year ago that might involve recovery), be a potential contraindication to safety (e.g., epilepsy), or involve medication use that may interfere with treatment response (i.e., bipolar disorder, schizophrenia).

**Headcap Positioning for HD-tDCS**

The headcap was positioned for each session using FPz and Cz as landmarks by measuring the nasion-inion and interaural lines.

**Clinical Information Collected**

Clinical data included age of AD diagnosis, use of medications for AD symptoms, and the Everyday Cognition scale.^1^ The Everyday Cognition scale is an informant-rated questionnaire of functioning in six areas: memory, language, visuospatial, planning, organization, and divided attention. A total score was computed by averaging the sum of ratings for all completed items, which provides a global measure of everyday cognitive function that ranges from 1-4 and correlates with cognitive stages.^1,2^ This scale was used to characterize AD stage for the sample at baseline.

**Method of Deriving Cognitive Scores and Test Forms Used**

Standardized scores were derived from published test manuals, and converted to T scores when needed for consistency across measures. Alternate versions were used for the RAVLT, BVMT-R, and BNT-SF. RAVLT versions consisted of the Taylor 1959,^3^ Geffen 1994,^4^ and Majdan 1996^5^ forms. BVMT-R versions consisted of forms 1, 2, and 3. BNT-SF consisted of administration of odd and then even item versions, with the odd item version used at both baseline and the 2-month follow-up.

**Method of Evaluating 8-week Follow-up Data**

Pairwise comparisons were used to evaluate if scores at the 8-week follow-up statistically differed between HD-tDCS conditions (sham versus 1mA; sham versus 2mA), while covarying for baseline performance. The resulting effect sizes were also transformed to Cohen’s *d* for further examination.

**HD-tDCS Tolerability**

Participants were administered a questionnaire^6^ to assess for side-effects during and after each HD-tDCS session. The incidence of reported side-effects was similar among the 3 HD-tDCS conditions (Table 2). Tingling (88-100%), itching (44-60%), and burning sensations (33-55%) on the scalp were commonly reported during stimulation and mostly graded as mild (31% of these rated as moderate and 0% as severe).

**e-Table 1 Stimulation Side Effects and Tolerability**

|  | **Sham (n=5)** | |  | **1 mA (n=9)** | |  | **2 mA (n=9)** | |
| --- | --- | --- | --- | --- | --- | --- | --- | --- |
| **Symptom** | **Yes:No** | **%** |  | **Yes:No** | **%** |  | **Yes:No** | **%** |
| Headache | 0:5 | 0% |  | 0:9 | 0% |  | 1:8 | 11% |
| Neck pain | 0:5 | 0% |  | 0:9 | 0% |  | 0:9 | 0% |
| Scalp pain | 0:5 | 0% |  | 1:8 | 11% |  | 0:9 | 0% |
| Tingling | 4:1 | 80% |  | 9:0 | 100% |  | 8:1 | 88% |
| Itching | 3:2 | 60% |  | 4:5 | 44% |  | 4:5 | 44% |
| Burning sensation | 2:3 | 40% |  | 3:6 | 33% |  | 5:4 | 55% |
| Skin redness | 3:2 | 60% |  | 2:7 | 22% |  | 3:6 | 33% |
| Drowsiness | 0:5 | 0% |  | 3:6 | 33% |  | 2:7 | 22% |
| Concentration difficulty | 1:4 | 20% |  | 0:9 | 0% |  | 0:9 | 0% |
| Acute mood change | 0:5 | 0% |  | 0:9 | 0% |  | 0:9 | 0% |

| **e-Table 2 Measurement Variability on Cognitive Outcomes** | | | | | | | | | | | | |
| --- | --- | --- | --- | --- | --- | --- | --- | --- | --- | --- | --- | --- |
|  | **Sham** | |  | **1 mA** | | | |  | **2 mA** | | | |
| **Measures** | **SD Time 1** | **SD Time 2** |  | **SD Time 1** | **SD Time 2** | **Adj SE**  **T2 vs sham** | |  | **SD Time 1** | **SD Time 2** | **Adj SE**  **T2 vs sham** |  |
| RAVLT total learning | 10.4 | 11.7 |  | 11.2 | 9.7 | 3.3 |  |  | 10.3 | 12.8 | 4.6 |  |
| RAVLT delayed recall | 0.9 | 1.8 |  | 6.1 | 6.2 | 1.0 |  |  | 2.7 | 1.8 | 0.9 |  |
| BVMT-R total learning | 1.1 | 2.6 |  | 8.9 | 5.2 | 1.7 |  |  | 3.1 | 2.0 | 1.6 |  |
| BVMT-R delayed recall | 3.1 | 1.3 |  | 12.7 | 10.1 | 2.5 |  |  | 1.0 | 5.2 | 2.4 |  |
| BNT-SF | 16.2 | 14.9 |  | 8.1 | 9.0 | 2.6 |  |  | 9.0 | 8.1 | 2.6 |  |
| DKEFS phonemic fluency | 13.7 | 14.9 |  | 8.7 | 9.7 | 4.0 |  |  | 14.5 | 14.2 | 4.0 |  |
| DKEFS category fluency | 10.6 | 15.7 |  | 6.4 | 8.7 | 3.4 |  |  | 6.0 | 5.6 | 3.3 |  |
| DKEFS category switching | 3.1 | 5.1 |  | 8.2 | 10.9 | 3.6 |  |  | 6.9 | 8.1 | 3.5 |  |
| TMT A | 16.9 | 17.2 |  | 13.8 | 17.0 | 4.4 |  |  | 16.6 | 16.4 | 4.4 |  |
| TMT B | -- | -- |  | -- | -- | -- |  |  | -- | -- | -- |  |
| SWAPS | 7.7 | 10.1 |  | 14.0 | 10.0 | 2.7 |  |  | 5.5 | 8.8 | 2.6 |  |
| DKEFS Color Naming | 12.1 | 18.4 |  | 9.7 | 10.6 | 5.3 |  |  | 7.4 | 12.0 | 5.2 |  |
| DKEFS Word Reading | 15.5 | 14.5 |  | 12.5 | 14.1 | 6.5 |  |  | 12.8 | 16.3 | 6.5 |  |
| DKEFS Inhibition | -- | -- |  | -- | -- | -- |  |  | -- | -- | -- |  |
| SD = standard deviation. Adj SE = standard error from general linear models comparing post-treatment scores (Time 2; T2) between active HD-tDCS condition and sham adjusted for pre-treatment scores (Time 1). RAVLT = Rey Auditory Verbal Learning Test. BVMT-R = Brief Visuospatial Memory Test-Revised. BNT-SF = Boston Naming Test 30-item Short Form (odd and even). DKEFS = Delis-Kaplan Executive Function System. TMT = Trail Making Test. SWAPS = Southwestern Assessment of Processing Speed. | | | | | | | | | | | | |

**e-References**

1. Farias ST, Mungas D, Reed BR, et al. The measurement of everyday cognition (ECog): scale development and psychometric properties. *Neuropsychology.* 2008;22(4):531-544.

2. Farias ST, Mungas D, Harvey DJ, Simmons A, Reed BR, DeCarli C. The measurement of everyday cognition: development and validation of a short form of the Everyday Cognition scales. *Alzheimer's & Dementia.* 2011;7(6):593-601.

3. Taylor EM. Psychological appraisal of children with cerebral defects. Cambridge, MA:Harvard University Press;1959: 423-428.

4. Geffen G, Butterworth P, Geffen LB. Test-retest reliability of a new form of the auditory verbal learning test (AVLT). *Archives of Clinical Neuropsychology.* 1994;9:303-316.

5. Majdan A, Sziklas V, Jones-Gotman M. Performance of healthy subjects and patients with resection from the anterior temporal lobe on matched tests of verbal and visuoperceptual learning. J*ournal of Clinical and Experimental Neuropsychology*, 1996;18:416-430.

6. Brunoni AR, Amadera J, Berbel B, Volz MS, Rizzerio BG, Fregni F. A systematic review on reporting and assessment of adverse effects associated with transcranial direct current stimulation. *International Journal of Neuropsychopharmacology.* 2011;14(8):1133-1145.
