# Supplementary material for: High definition transcranial direct current stimulation as an intervention for cognitive deficits in Alzheimer's dementia: A randomized controlled trial
Source: J Prev Alzheimers Dis. 2025 Jan 1;12(2):100023. doi: 10.1016/j.tjpad.2024.100023 (PMC11895854; doi:10.1016/j.tjpad.2024.100023)
Supplement: Supplementary file 2 [file mmc2.docx]

**Protocol**

**HD-tDCS as an intervention for cognitive deficits in Alzheimer’s dementia: a randomized controlled trial**

Document History:

Version Date Author Notes

0.1 October 21, 2021 C LoBue Original draft

1 April 28, 2022 C LoBue Modified battery of

neuropsychological measures to replace with more comprehensive versions of measures. Included the RAVLT, DKEFS Verbal Fluency, and DKEFS Color-Word Interference test. Corrected written follow-up visit timeline to align with timing of visits expected by study sponsor.

Version: Final

Signature _________________

Principal Investigator: Christian LoBue, PhD

**Sponsor:** Alzheimer’s Women’s Association for Resources and Education (AWARE),

the Dallas Foundation

**Title:** Noninvasive Brain Stimulation in Mild Cognitive Impairment and Dementia

**Trial registration:** ClinicalTrials.gov; NCT05270408

**Trial design:** randomized, double-blind, parallel-group sham-controlled trial

**Number of arms:** 3

**Trial site:** University of Texas Southwestern Medical Center

**Protocol ID:** STU-2021-0974

**Participant time in trial:** Two months

**Background:**

The Alzheimer’s Clinical syndrome (ACS) involves the stages of mild cognitive impairment (MCI) and dementia. While recent disease-modifying treatments for Alzheimer’s disease show promise to slow cognitive decline, these do not target the cognitive deficits present and are unproven to lessen symptoms. Interventions that can target the damage to memory circuitry in individuals with MCI as well as Alzheimer’s dementia (AD) to improve episodic memory is vital to fill an unmet need. Noninvasive neuromodulation therapies have been found to have promising benefits for treating depression and improving cognitive function in patients with and without brain lesions. Specifically, high-definition transcranial direct current stimulation (HD-tDCS) uses an array of small electrodes to pass a low level current through targeted brain regions (e.g., anterior cingulate cortex) to modulate neuronal activity and brain circuitry. HD-tDCS is a safe, portable, and low-cost intervention that offers excellent potential to lessen symptoms in patients with MCI and AD.

**Purpose:**

This is a pilot study being done to attempt to improve verbal episodic memory in persons with MCI and AD. Although the hippocampus is a brain structure most often associated with early deficits in MCI and dementia, the dorsal anterior cingulate cortex (DACC) has been shown to play a role in verbal episodic memory for such patients. Delivery of HD-tDCS to the DACC may have potential to entrain the circuit involved in episodic memory, and as such, may improve the functioning of the circuit and lessen symptoms. The purpose of the pilot study is to examine the efficacy of HD-tDCS to the DACC region and its influence on verbal episodic memory in patients with MCI and dementia.

**Primary Objective:**

- Determine if HD-tDCS lessens memory deficits​ associated with ACS

**Secondary Objective:**

- Determine if HD-tDCS enhances language, processing speed, and executive functions in ACS
- Evaluate if cognitive changes produced from HD-tDCS persist for 8 weeks

**Design:**

Participants with a clinical diagnosis of AD as well as MCI will be enrolled. Participants will receive 10 sessions of 1 mA , 2 mA, or sham HD-tDCS stimulation across 2 weeks. Each HD-tDCS session will be 20 minutes in duration and have a 4x1 ring configuration for electrode placement (anode: Fz; cathodes at FPz, Cz, F7, and F8) to deliver stimulation to the DACC. Cognitive assessments will be completed at a baseline visit, immediately following the last HD-tDCS session, and again at a 2-month follow-up visit.


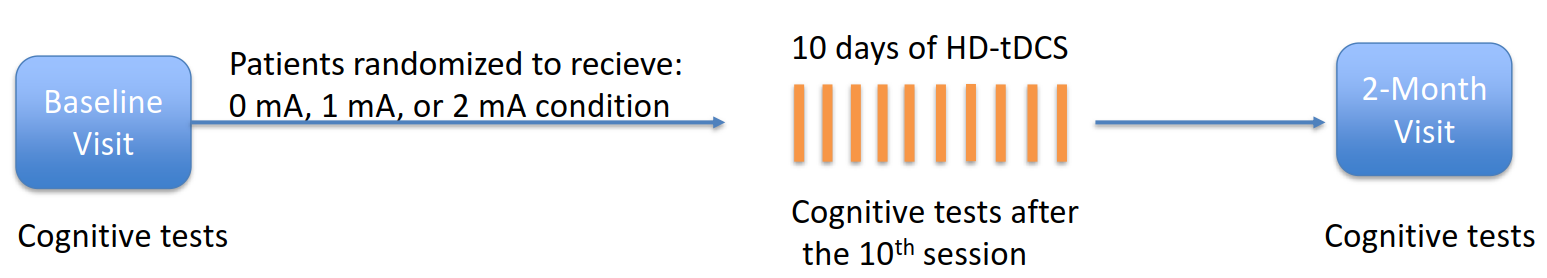


| **SUMMARY OF VISITS** | | |
| --- | --- | --- |
| **Visit** | **PROCEDURES** | **TIME** |
| Visit 1 | Pre-testing neuropsychological assessment | 1 hour |
| Visits 2-10 | HD-tDCS active or sham stimulation | 30 minutes |
| Visit 11 | HD-tDCS active or sham stimulation and post-testing neuropsychological assessment | 1 hour 30 minutes |
| Visit 12 | 8-week follow-up assessments | 1 hour |

**Inclusion criteria:**

1. Age 50 and older
2. Fluent in English
3. Active diagnosis of MCI or Alzheimer’s dementia

**Exclusion criteria:**

1. Substance use disorder within past year
2. Has metal fragments or implants in head
3. Traumatic brain injury within past year
4. Lifetime major medical that could be a contraindication to safety (e.g., epilepsy)
5. Lifetime psychiatric conditions (e.g., bipolar disorder, schizophrenia) require taking medications that may interact with the HD-tDCS effect (e.g., carbamazepine, sulpiride, lorazepam)

**Procedures:**

**HD-tDCS arms of intervention:** The study has 3 arms of intervention. The HD-tDCS treatment uses a cap with arrays of electrodes, combinations of which can be optimized for targeting delivery of electrical current to specific brain regions. HD-tDCS is delivered with sintered 12 mm diameter Ag/AgCl disc electrodes. The EEG cap is gently secured on the head of a subject and positioned with Cz at the vertex, as measured using surface anatomical landmarks, and defined as the intersection of the nasion–inion and interaural lines. Electrical stimulation will be performed while away and at rest using a constant voltage stimulator manufactured by Neuroeletrics (Starstim system). Electrodes will be filled with a conductive gel. Contact quality and impedance levels (< 10 kOhms) will be verified for each electrode before each stimulation session begins. HD-tDCS will be delivered for each arm on separate days for 10 sessions over 10-14 days. After each session, patients will be administered a standard post-treatment symptom questionnaire (e.g., assessing for skin irritation, fatigue).

Arm 1: For the 1 mA active HD-tDCS condition, administration parameters will consist of the stimulation being ramped up over 60 seconds until it reaches 1 mA and then maintained for 20 minutes. The anode will be placed over Fz according to the International 10-20 EEG system, corresponding to the approximate location of the DACC. Four return cathodal electrodes will be placed approximately 5 cm radially from Cz; corresponding to locations Fz, FPz, F7, F8 and Cz.

Arm 2: For the 2 mA active HD-tDCS condition, administration parameters will consist of the stimulation being ramped up over 60 seconds until it reaches 1 mA and then maintained for 20 minutes. The anode will be placed over Fz according to the International 10-20 EEG system, corresponding to the approximate location of the DACC. Four return cathodal electrodes will be placed approximately 5 cm radially from Cz; corresponding to locations Fz, FPz, F7, F8 and Cz.

Arm 3: For the sham condition, parameters will consist of the current being ramped up over 60 seconds until it reaches 1 mA and then stopped. This allows for the same scalp sensation for both the sham and active condition so that subject and experimenter will remain blinded. This ramp up for one minute and then stopped and off for 20 minutes will be given each 20-minute sham session to keep the timing and subjective experience similar to the active condition.

All 3 HD-tDCS conditions will be pre-programmed into the Neuroelectrics software prior to study initiation. Conditions will be labeled with a non-descriptive name that included the study name and a protocol number (1-3). Aside from the PI, other study team members will be unaware of how the HD-tDCS conditions were labeled in order to preserve blinding.

**Randomization:** Participants were randomized at a ratio of 1:2:2 according to the sham, 1 mA, and 2 mA conditions. Randomization of HD-tDCS condition assignments were generated by a computer, and the assignments (Protocol 1-3) recorded on cards and placed in sealed envelopes by the PI.

**Blinding:** Participants will be informed that sham and active HD-tDCS will be used in the study, but they will be blinded to condition. Aside from the PI, other study team members who perform assessments will be blinded to HD-tDCS condition, and the subject and study personnel will complete a questionnaire to check if blinding was successful.

**Sample size calculation:** No power analysis was performed for the study given the pilot nature of the trial.

**Recruitment strategy:** Participants will be recruited from the Neurology and Neuropsychology ambulatory clinics at the University of Texas Southwestern Medical Center (UTSW) as well as Baylor Medical Center’s AT&T Memory Center. Clinician referrals and self-referrals will be used to identify subjects directly for screening/recruitment. Strategies for obtaining these will include 1) providing study-related information with colleagues at selected Clinics via e-newsletters and university presentations, 2) placing flyers in exam rooms and waiting areas at clinic facilities, 3) advertising on the UTSW website, and 4) outreach to regional community sources.

**Screening and consenting strategy:** Prospective participants will undergo telephone screening for conditions in their medical history as well as current medications. Prospective participants meeting pre-screening inclusion/exclusion criteria will be scheduled an appointment to review the study procedures and Consent Form. Study staff will go over the consent form in detail with the prospective participant and answer any questions about the procedures. Participants or the legal authorized representative will be asked to sign the Consent Form if they understand the study procedures, appreciate potential risks/benefits, and desire to be involved in the study.

**Timing and procedures of follow-up:** Cognitive evaluations will be completed immediately after session 10 and 2-months later. Patients will be called back for the 2-month follow-up.

**Outcomes:**

**Primary**

Episodic memory functioning will be assessed through standardized scores in Total Learning and Delayed Recall for the Rey Auditory Verbal Learning Test (verbal memory).

**Secondary**

- Visual memory will be assessed through standardized scores in Total Learning and Delayed Recall for the BVMT-R.
- Language will be assessed through standardized scores for the Boston Naming Test, DKEFS Phonemic Fluency, and DKEFS Category Fluency.
- Processing speed will be assessed through standardized scores for the SWAPS, TMT A, DKEFS Color Naming, and DKEFS Word Reading.
- Executive functions will be assessed through standardized scores for TMT B, DKEFS Category Fluency Switching, DKEFS Inhibition, DKEFS Inhibition/Switching.

Rey Auditory Verbal Learning Test (RAVLT). A 15-item list learning and memory task where the tester reads aloud a list of nouns. The RAVLT includes 5 learning trials, an interference trial, a delayed recall trial, and a yes/no delayed recognition trial. The tester records how many items the patient remembers over several repeated trials and then again for the interference and 30-minute delayed recall trial. The RAVLT is a direct measure of verbal episodic memory and has multiple alternate forms. The scores forTotal Learning and Delayed Recall are the outcomes of interest.

Brief Visuospatial Memory Test-Revised (BVMT-R). A three trial figural learning and memory task where the tester shows the patient an 8 x 11-inch array containing 6 simple geometric visual designs in a 2 x 3 matrix for 10 seconds. Patients are asked to reproduce as many designs as possible in the same location as they appeared on the display for each trial. A delayed recall trial is completed after 25 minutes followed by a yes/no recognition trial. The tester scores each trial (Learning and Delayed Recall) in terms of the accuracy and location of each design reproduced by the patient. The BVMT-R is a measure of visual episodic memory and has 6 alternate forms. The scores for Total Learning and Delayed Recall are the outcomes of interest.

Delis-Kaplan Executive Function System (DKEFS) Phonemic Fluency. This is an assessment of word retrieval during which the patient is required to produce as many words in one minute that begin with a specific letter (e.g., *F, A,* or *S*). The outcome measure for this task is the total number of correct responses across the trials.

DKEFS Semantic Fluency. This is an assessment of word retrieval that involves 3 conditions. The patient is required to produce as many words in one minute that are members of specific categories (e.g., condition 1 = *animals*, condition 2 = *boy’s names*) or switching between two different categories (condition 3 = fruits-and-furniture items). The outcome measures for this task are the total words named for the two semantic conditions and the total words named during the switching condition.

Boston Naming Test – Short Form. This is an assessment of word retrieval during which a 30 item version of the task (odd and even item versions of the full test) is administered. The subject is shown pictures of objects and is required to correctly name them as quickly as possible. The outcome measure for this task is the total number of correct responses.

Trail Making Test. This is an assessment with 2 conditions. Trails A measures processing speed and simple attention, while Trails B utilizes the same cognitive components as Trails A with the additional executive function of alternating sequencing. The tasks require the subject to as quickly as possible complete both conditions, involving number sequencing and then number-letter switching. The outcome measure for this task is the time to complete each of the two conditions.

DKEFS Color-Word Interference. This test measures processing speed in the control conditions of word reading and color naming, measures executive functions of inhibitory control in the interference condition, and cognitive flexibility in the switching condition. The interference condition is where the color word names are written in a different ink color than the name. The switching condition is where the patient must switch back-and-forth between the response patters of naming the ink color and reading the word. The outcome measure for this task is the time to complete each of the four conditions.

Southwestern Assessment of Processing Speed (SWAPS). This is an assessment of processing speed. The patient is required to transcribe numbers to their corresponding written symbol as quickly as possible within 60 seconds. The outcome measure for this task is the total number of correct responses.

**Subject safety monitoring:** All HD-tDCS sessions will be overseen by the PI. All safety precautions recommended by scientific literature and the UT Southwestern IRB will be carefully followed. All adverse events volunteered by the subject or elicited by the research team will be recorded in the subjects’ research file. All adverse events will be reported within one week to the IRB. Any severe adverse events will be reported within 24-48 hours to the IRB.

In order to safeguard our participants from a breach in confidentiality, the data is coded with a unique semi-random subject identifier (URSI). All data after initial entry into the study is coded based on the participant’s number. A digital file containing completed questionnaires and assessments from the experimental session will be maintained with the URSI number. These files will be kept in an encrypted, IRB approved database (RedCap).

**Statistical analysis:** General linear models will be used to evaluate whether outcomes significantly change as a function of treatment allocation (sham, 1 mA HD-tDCS, 2 mA HD-tDCS). Changes from baseline on the outcomes of interest will be assessed. Participants with missing time points will not be excluded. All available data on all participants who 1) meet inclusion/exclusion criteria, 2) have been randomly assigned to groups, and 3) provide baseline data will be included regardless of whether they complete the trial.
