# Supplementary material for: High definition transcranial direct current stimulation as an intervention for cognitive deficits in Alzheimer's dementia: A randomized controlled trial
Source: J Prev Alzheimers Dis. 2025 Jan 1;12(2):100023. doi: 10.1016/j.tjpad.2024.100023 (PMC11895854; doi:10.1016/j.tjpad.2024.100023)
Supplement: Supplementary file 3 [file mmc3.docx]

**Statistical Analysis Plan**

**HD-tDCS as an intervention for cognitive deficits in Alzheimer’s dementia: a randomized controlled trial**

Document History:

Version Date Author Modifications

0.1 March 4, 2024 C LoBue Original draft

0.1 March 14, 2024 A Salter Review

0.2 March 14, 2024 C LoBue Added sections: design,

randomization, sample size calculation, and outcomes. Accepted changes in re-arrangement of analysis details

0.3 March 27, 2024 C LoBue Modified analysis

methods as follows. Changed effect size calculation from the pooled pretest standard deviation approach (dppc2) to use of Cohen’s *d*. Changed from use of Cochran-Armitage test to Chi-square/Fisher’s exact tests in evaluating significance of proportions of clinically meaningful change.

1 July 8, 2024 C LoBue Updated. Removed Chi-

square/Fisher’s exact test from plan.

Version: Final

Signature _________________ 8/12/2024

Principal Investigator: Christian LoBue, PhD

**Contents:**

**Objectives 3**

**Design 3**

**Randomization 3**

**Blinding 3**

**Study sample 3**

**Sample size calculation 3**

**Outcomes 4**

**Analysis methods 4**

**Objectives:**

- Determine if HD-tDCS lessens Alzheimer’s dementia (AD) associated memory deficits​
- Determine if HD-tDCS enhances language, processing speed, and executive functions in AD
- Evaluate if cognitive changes produced from HD-tDCS persist for 8 weeks

**Design:**

Participants with a clinical diagnosis of AD or mild cognitive impairment were enrolled. Participants received 10 sessions of 1 mA , 2 mA, or sham HD-tDCS stimulation across 2 weeks. Each HD-tDCS session was 20 minutes in duration and had a 4x1 ring configuration for electrode placement (anode: Fz; cathodes at FPz, Cz, F7, and F8) to deliver stimulation to the dorsal anterior cingulate cortex based on its role in episodic memory functioning and other cognitive skills such as language and executive functions. Cognitive assessments were completed at a baseline visit, immediately following the last HD-tDCS session, and again at a 2-month follow-up visit.


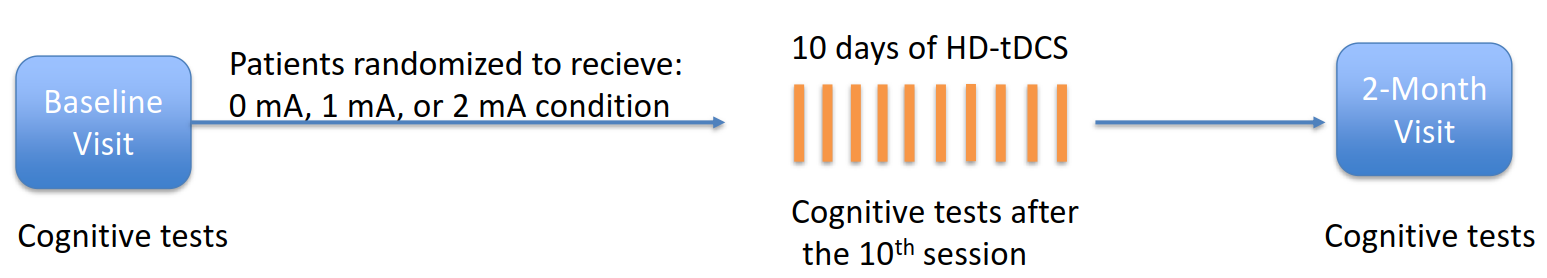


**Randomization:**

Participants were randomized at a ratio of 1:2:2 according to the sham, 1 mA, and 2 mA conditions. Randomization of HD-tDCS condition assignments were generated by a computer, and the assignments recorded on cards and placed in sealed envelopes by the PI.

**Blinding:**

Participants will be informed that sham and active HD-tDCS will be used in the study, but they will be blinded to condition. Aside from the PI, other study team members who perform assessments will be blinded to HD-tDCS condition, and the subject and study personnel will complete a questionnaire to check if blinding was successful.

**Study sample:**

The study sample will be limited to participants with a clinical diagnosis of AD.

**Sample size calculation:**

No power analysis was performed for the study.

**Outcomes:**

**Primary**

Episodic memory functioning will be assessed through standardized scores in Total Learning and Delayed Recall for the RAVLT (verbal memory).

**Secondary**

- Visual memory will be assessed through standardized scores in Total Learning and Delayed Recall for the BVMT-R.
- Language will be assessed through standardized scores for the Boston Naming Test, DKEFS Phonemic Fluency, and DKEFS Category Fluency.
- Processing speed will be assessed through standardized scores for the SWAPS, TMT A, DKEFS Color Naming, and DKEFS Word Reading.
- Executive functions will be assessed through standardized scores for TMT B, DKEFS Category Fluency Switching, DKEFS Inhibition, DKEFS Inhibition/Switching.

**Changes to outcomes:** There were no changes to outcomes after the trial commenced.

**Analysis Methods:**

Two pairwise comparisons will be used to evaluate whether cognitive performance following the last treatment session statistically differs between HD-tDCS conditions, covarying for pre-treatment scores. This will consist of sham versus 1 mA and then sham versus 2 mA conditions. Eta^2^ effect sizes produced from the two comparisons will be transformed to Cohen’s d and examined.

Clinically meaningful change associated with HD-tDCS will also be assessed. Proportions of individuals with a 5+ standardized score increase (T-score) on each measure of cognitive functioning will be characterized and evaluated. This will be completed examining sham versus 1 mA and then sham versus 2 mA conditions.

For the exploratory objective, those outcome measures identified to show significant changes immediately following the last treatment session (via statistical or Cohen’s *d* ≥ .50) will be evaluated for this aim. Pairwise comparisons will be used to evaluate if scores at the 8-week follow-up statistically differ between HD-tDCS conditions (sham versus 1 mA; sham versus 2 mA), while covarying for the pre-treatment scores. Eta^2^ effect sizes produced from comparisons will be transformed to Cohen’s d and examined.

**Missing data:**

In the event few scores of executive function are available (due to tests being discontinued from cognitive impairment), alternative indices will be derived and examined post-hoc. Number of items not completed when the time limit is reached will be summed (higher scores representing lower performance) for TMT B, DKEFS Inhibition, and DKEFS Inhibition/Switching measures. Scores will be examined in ANCOVA models and effect sizes, but not as indices for clinically meaningful change.

**Multiple comparison correction:**

Statistical significance will be set at p < 0.05 with no correction for multiple comparisons to reduce the chances of a Type II error given the small sample size of the pilot study.
